# Invasive Cutaneous Candidiasis, Autoimmune Hemolytic Anemia and Pancytopenia: A Challenging Scenario for Waldenström Macroglobulinemia in an Elderly Patient

**DOI:** 10.3390/biomedicines11041007

**Published:** 2023-03-24

**Authors:** Juan Carlos Caballero, Elham Askari, Nerea Carrasco, Miguel Angel Piris, Begoña Perez de Camino, Laura Pardo, Javier Cornago, Jose Luis Lopez-Lorenzo, Pilar Llamas, Laura Solan

**Affiliations:** 1 Hematology Department, Fundacion Jimenez Diaz University Hospital, 28040 Madrid, Spain; juan.caballero@quironsalud.es (J.C.C.); easkari@quironsalud.es (E.A.); begona.perezc@quironsalud.es (B.P.d.C.); laura.pardo@quironsalud.es (L.P.); javier.cornago@hospitalreyjuancarlos.es (J.C.); jllopez@quironsalud.es (J.L.L.-L.); pllamas@fjd.es (P.L.); 2 Infectious Diseases Department, Fundacion Jimenez Diaz University Hospital, 28040 Madrid, Spain; ncarrascoa@fjd.es; 3 Pathology Department, Fundacion Jimenez Diaz University Hospital, 28040 Madrid, Spain; miguel.piris@quironsalud.es

**Keywords:** Waldenström macroglobulinemia, invasive candidiasis, granulomatosis, case report

## Abstract

Waldenström macroglobulinemia (WM) is a slowly progressive hematologic malignancy that usually responds rapidly to treatment. Being a lymphoplasmacytoid neoplasm, it is associated with a monoclonal IgM component, which may be associated with multiple manifestations and symptoms. We report the case of a 77-year-old woman diagnosed with WM following the development of severe and sudden pancytopenia associated with a cold agglutinin syndrome. In order to treat the WM and the underlying hemolysis, treatment with rituximab, corticosteroids and cyclophosphamide was started. Despite the improvement in hemolysis parameters, pancytopenia persisted, and we started a second line with ibrutinib. During treatment the patient developed an uncommon invasive fungal infection (IFI) with bone marrow granulomatosis and myelofibrosis. This case shows an unusual clinical course with a poor hematopoietic response to treatment and a large number of intercurrent complications.

## 1. Introduction

Waldenström macroglobulinemia (WM) is a B-cell lymphoproliferative syndrome (LPS), defined by the infiltration of small lymphocytes as well as lymphoplasmacytoid cells and plasma cells in the bone marrow (BM), producing a detectable monoclonal immunoglobulin M (IgM) [1].

According to the World Health Organization (WHO), the underlying disorder is a lymphoplasmacytic lymphoma (LPL) [2], where MYD88 mutation is present in more than 90% of cases, and this and other recent advances have enhanced our understanding [3].

Since other lymphomas present a faster clinical presentation, LPL usually describes a long clinical course reaching an overall survival of nearly 7 years [4]. Thus, a considerable number of cases benefit from monitoring without any therapy [5]. Nevertheless, 75–85% of all cases may require treatment based on the development of symptoms derived from the disease [6].

As a result, manifestations may vary depending on the involved tissue, including BM or extramedullary infiltration, or those related to the monoclonal component [6]. Therefore, IgM paraprotein can cause specific complications due to its physical–chemical properties, autoantibody activity, tissue deposition and non-specific interactions with other proteins, such as cryoglobulins, cold agglutinin syndrome (CAS) and amyloidosis [7].

The reason to report this case is the unusual, abrupt and severe course of a commonly silent disease. The appearance of pancytopenia due to BM infiltration combined with hemolytic anemia and complicated by an invasive fungal infection with BM granulomatosis and skin involvement (only seen in up to 5% of IFIs) makes this an atypical and unique case [8].

## 2. Patient Background

A 77-year-old woman arrived at the emergency department with fever and abdominal pain. The patient was diagnosed in 2002 with ulcerative colitis controlled with mesalazine and bullous pemphigoid, and around 2010 managed with topical corticosteroids. In 2019 the patient was diagnosed with IgM monoclonal gammopathy with no signs or symptoms of systemic involvement, and not meeting the criteria for further study or treatment [7]. No further relevant background information was documented.

A month prior to admission at our center, the patient was studied at her home hospital for progression of her hematologic disease due to progressive cytopenia. According to this, a BM biopsy was performed, and she was diagnosed with a low-grade LPS. Treatment with four doses of weekly rituximab was started. After this, no further studies had been made at her home hospital.

## 3. At Our Hospital

On first approach, she showed progressive jaundice, asthenia, anorexia, weight loss, fever and dyspnea. Blood tests suggested hemolytic anemia with 61 g/L hemoglobin (120–150), 45 × 10^9^/L platelets (150–450), 3 mg/dL bilirubin (0.3–1.2), 475 U/L LDH (inferior 250) and undetectable haptoglobin, with a white blood cell count of 49 × 10^9^/L (3.5–12) with 39 × 10^9^/L (1.2–5.0) lymphocytes. Additionally, a peripheral blood (PB) smear showed anisopoikilocytosis, dacryocytes and spherocytes. Finally, a direct Coombs polyspecific test had a positive result for complement with 1/1024 dilution and IgG of 1/2 dilution. Due to these assessments, she was diagnosed with mixed hemolytic anemia. Therefore, treatment with corticosteroids at a dose of 1 mg/kg/day was initiated as an inpatient. As follow-up, study was intensified with specific methods, showing strong positivity for complement C3d fraction and the presence of anti-Yta irregular antibody. A diagnosis of mixed autoimmune hemolytic anemia (AIHA) was made, not being able to classify it as cold agglutinin disease (CAD) or syndrome (CAS) due to the inability to perform a cold agglutinin titer. Hence, corticosteroids were withdrawn. After resolution and when necessary, only phenotypically identical red blood cells were transfused.

In addition, given this scenario, different possibilities were considered. Subsequent diagnostic studies were performed to rule out systemic infections, transformation of her LPS to a high-grade lymphoma or progression of the lymphoplasmacytic lymphoma.

## 4. The Hematologic Malignancy Diagnosis

Among all the diagnostic tests performed on the patient, the following stood out: a body computerized tomography (CT) scan was requested, showing an 18 cm splenomegaly. Moreover, all other tests were as listed: serum protein electrophoresis revealed 2438 g/dL of monoclonal IgM with 1.48 g/dL of M component; the free light chain (FLC) resulted in being lambda with a 0.24 (0.26–1.65) FLT ratio; the PB immunophenotype showed the expression of B LPS with an unclassifiable immunophenotype; and 60% of the total leukocytes expressed CD45, CD19, CD20, CD22, CD25 and lambda surface immunoglobulin, with 0.55% of the cells showing lymphoplasmacytoid differentiation.

On top of everything listed above, the BM biopsy showed extensive infiltration (>75%) by monoclonal lymphoplasmacytes and small plasma cells with lambda restriction. These showed B phenotype (CD20+ and CD79+) and BCL2 expression. No expression of BCL6, CD10 or cyclin D1 was observed. Lastly, molecular testing revealed MYD88^L265P^ mutation. Due to all the evidence here presented, a diagnosis of LPL was established (Table 1).

## 5. Treatment Choice

Our patient had received first line treatment with four doses of weekly rituximab without response. That being the case, a short course of oral cyclophosphamide at 200 mg/day dose for 5 days was started to control hemolytic anemia and the underlying disease.

Within two weeks of treatment, hemolysis was controlled with the normalization of bilirubin, haptoglobin and LDH values and an improvement in her dyspnea, fatigue and jaundice. Nevertheless, the severe pancytopenia was not controlled. With the intention of improving pancytopenia, a new line of treatment was proposed to control the underlying disease. As a result, the two best options in the case of our patient, and according to the guidelines [9], were rituximab plus bendamustine or ibrutinib. Given our patient’s pancytopenia due to BM infiltration, we decided to start treatment with a daily dose of ibrutinib 420 mg [10].

## 6. Infectious Complications

One week after ibrutinib’s introduction, she developed febrile neutropenia. Therefore, cefepime at 6 g/day was started. The microbiology department isolated *Candida tropicalis* from blood cultures. Hence, caspofungin was started to control a probable IFI.

**Figure 1:**
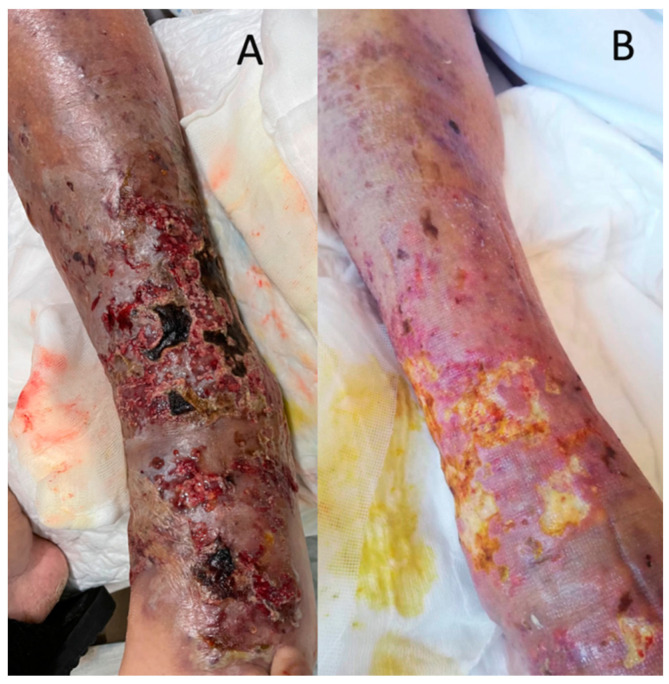
(**A**) Exudative necrohemorrhagic lesions located in the lower third of the left leg. Patchy involvement can be observed on healthy skin with a tendency to violaceous coloration. (**B**) Evolution of cellulitis lesions in left leg after systemic treatment with caspofungin. Disappearance of necrohemorrhagic lesions and improvement in color and appearance of the surrounding skin can be observed.

**Figure 2:**
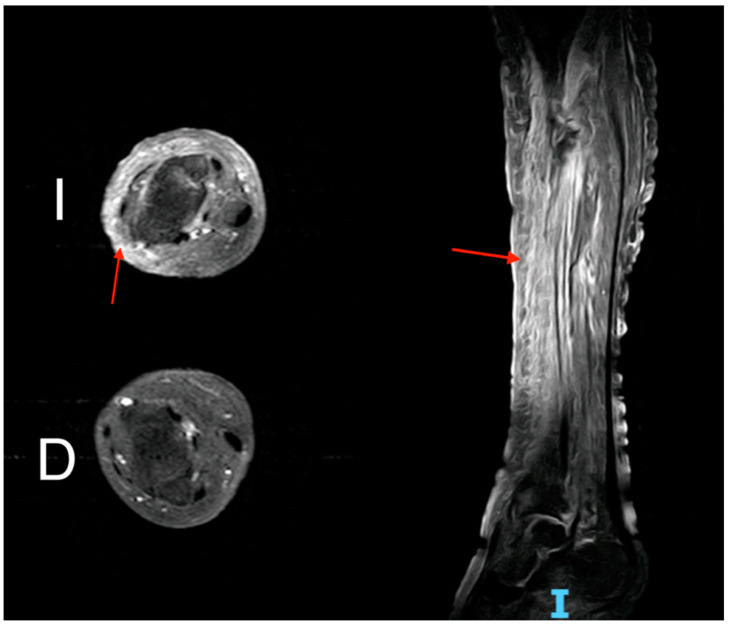
Magnetic resonance imaging of legs in axial (**left**) and sagittal (**right**) views. Arrows show the deep involvement of the infection with hyperintense image.

Simultaneously, the patient started to have pain and swelling in her left leg. Deep venous thrombosis was dismissed with a doppler echography. Nonetheless, Gram-positive coverage with Clindamycin 2400 mg/day was started with worsening evolution. Ulcerative exudative lesions appeared with patchy necrosis (Figure 1 and Figure 2).

**Figure 3:**
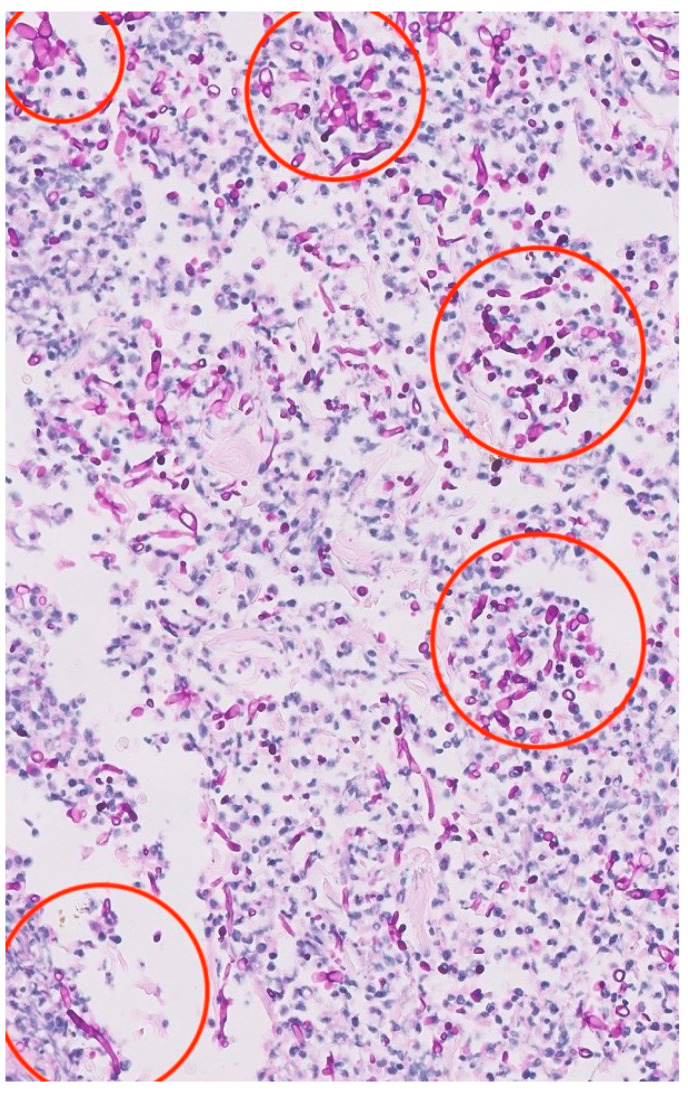
Circled, pseudohyphae observed in skin sample of the lesions on the left leg. PAS staining (60×).

Taking into account the worsening situation and recent lesions, the dermatology, plastic surgery, and infectious diseases departments were consulted. A differential diagnosis included pyoderma gangrenosum secondary to ibrutinib and fungal myositis. Thus, a skin biopsy was taken and double antifungal coverage with caspofungin and fluconazole was added to topic treatment with corticosteroids. Finally, pathologists found “pseudohyphae” in line with blood cultures (Figure 3). After clinical improvement in the skin lesions (Figure 1), oral treatment with fluconazole was continued.

**Figure 4:**
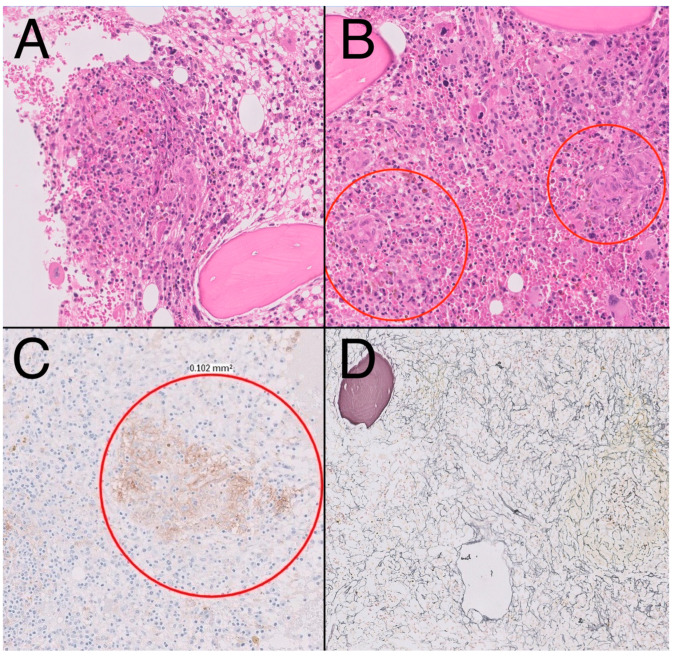
Histology of bone marrow. Circled granulomas are shown in different stains: hematoxylin–eosin (20×) (**A**,**B**) and CD11c (10×) (**C**). Reticulin staining showing grade 2 bone marrow fibrosis (10×) (**D**).

Moreover, ibrutinib was maintained for 3 weeks. With no evidence of hemolysis, pancytopenia persisted. A new BM study was performed showing less than 15% of medullar LPL infiltration and highlighting the presence of non-necrotizing epithelioid granulomas distributed in the stroma. No microorganisms were found by PAS and GROCOTT staining. The pathology department demonstrated grade 2 BM fibrosis (Figure 4) where the presence of CXCR4 mutation was also detected.

The IgM level gradually decreased below 200 mg/dL (complete serum response) with persistent cytopenia and transfusion requirements. That being the case, treatment was stopped until hematologic recovery, continuing support with daily granulocyte colony-stimulating factor and weekly erythropoietin. Two months later, cytopenia had not improved and the patient suffered repeated bacteremia, dying after 4 months of severe aplasia.

## 7. Discussion

Aging is associated with a state of immunosenescence with a deficient response to environmental aggressions. Consequently, different factors such as infections, chronic antigenic stimulation or immune evasion can trigger a dysregulation that increases the risk of developing lymphoma [11]. This condition associated with the deficient bone marrow reserve of these patients can lead to a complex scenario [12].

We present an atypical course of a newly diagnosed elderly woman with LPL. To begin, WM generally describes a long clinical course with high overall survival (7 years for patients between 70–80 years old) [4]. However, our case corresponds to high risk based on the International Prognostic Scoring System for WM (5 over 6) and very high risk based on the “Revised International Prognostic Score System” (5 over 5) [5,13]. Anyway, the development of AIHA at the time of diagnosis limited the patient’s management.

To sum up, recent reviews suggest CAD is an IgG+, C3d+/− and IgM+/− AIHA with ≥64 cold agglutinin titer in which finding any underlying disorder is not possible. Although it has recently been described that many of these episodes are in fact associated with low-grade LPS [14,15], on the other hand, CAS refers to a group of AIHA secondary to infections and aggressive lymphomas among others [14,15]. According to some authors, antibodies may even function as tumor markers, disappearing with tumor response and reappearing at recurrences [16]. While the Coombs test was always positive in our case, the crossmatch test became negative with treatment and hemolysis resolution. What came across as atypical is the development of hemolysis even after receiving rituximab since, reviewing the literature, it is associated with a good response and low recurrence rate [17]. Thus, in our case, this aggressive debut forced the use of therapies beyond monotherapy with rituximab, associating corticosteroids and cyclophosphamide. Its immunosuppressive and antineoplastic effect probably facilitated hemolysis control [18,19]. Even so, cytopenia barely responded.

As stated previously, the presence of MYD88^L265P^ mutation is highly characteristic of WM (>90%), followed by the CXCR4^WHIM^ mutations (~35%) [20]. Its presence impacts greatly on the response depth, time to major response and progression free survival [10].

According to this, MYD88^L265P^ mutated/CXCR4 wild type obtain a better and faster response than patients with both mutations [20].

With a global efficacy varying from 70% to 95% [21], ibrutinib is the most effective single agent in WM. Moreover, it is active in both rituximab-sensitive and rituximab-refractory patients [9]. As published by S. P. Treon et al., the overall response in previously treated patients is as high as 90.5% [22]. Ibrutinib achieved in our patient a partial response with a progressive decrease in IgM levels despite the mutational profile. Despite this, and as previously reported, pancytopenia showed no improvement. Consequently, genetic analysis will probably become one of the routine initial studies for choice of treatment or refractory cases [3].

Parallel to that, an infectious complication of her leg complicated the patient’s management. Differential diagnosis included *Pyoderma Gangrenosum* secondary to ibrutinib treatment (10 cases/million/year) [23]. The characteristics of the lesion and its appearance after drug initiation led us to suspect this causal relationship. This complication is closely related to ulcerative colitis and WM [24,25]. Nevertheless, pathologists determined that IFI was more likely to occur. Eventually, severe neutropenia, immunosuppressive treatments and broad spectrum antibiotics provided its development [26]. Furthermore, ibrutinib, especially when associated with steroids, chemotherapy or immune modulator agents, increases IFI risk. Thus, it impairs innate immune cell functions affecting neutrophil functionality mainly during the first 6 months of treatment [27]. Regardless, the risk of this condition in patients with WM appears to be lower than in patients with chronic lymphocytic leukemia treated with Bruton’s tyrosine kinase inhibitors [28].

Fortunately, the incidence of acute disseminated candidiasis has been reduced since the introduction of fluconazole prophylaxis in 1990 [29], as it is a typical and serious complication that affects critical patients [29,30]. Despite the characteristic yeast-like growth of *Candida*, it was possible to observe the fungi’s pseudohyphae formations on the skin. This manifestation is only found in 5% of disseminated candidiasis [8].

Mortality can reach 47% of patients even with the Infectious Diseases Society of America (*IDSA*) echinocandin coverage recommendation [31]. Unexpectedly, and with our patient being an immunocompromised host, evolution was favorable. As recommended by the guidelines, treatment was switched to fluconazole with a good response [32]. Nonetheless, with hemolysis and the LPL response, and even having successfully overcome the infectious complication, the patient did not obtain hematopoietic response.

One of the unusual findings was the presence of granulomas on the last BM biopsy. These formations are frequently found on histiocytosis, IgG4 disease or sarcoidosis [33,34,35]. However, the relationship between granulomas and infectious diseases seems clear. Some case series place non-Hodgkin’s lymphomas as one of the most frequent causes for the development of granulomas, with no association with LPL [36,37]. The fact that granulomatous lesions were not found by the diagnostic BM biopsy (with massive LPL infiltration) in contrast with its finding on the sample with only 15% infiltration suggests that the most likely etiology was systemic infection caused by *Candida tropicalis.*

Likewise, the presence of grade 2 BM fibrosis, granulomas and systemic infection could worsen the patient’s hematopoietic recovery [38]. These three issues were probably the cause of a multifactorial pancytopenia. In contrast to this, according to the International Workshops on WM (IWWM-7) [39] response criteria, IgM decreased by 70%, which means there was a partial response.

## 8. Conclusions

This report highlights how MW can develop quickly and severely. A thorough knowledge of the disease and the in-depth analysis of LPL’s molecular biology can help us to choose the best treatment option and foresee a worse evolution. On the other hand, deep neutropenia and immunosuppressive therapy should make us suspect and treat a possible fungal disease with atypical presentation.

## Figures and Tables

**Table 1:** Analytical characteristics of the patient’s disease. BM evaluation, flow cytometry, histology and molecular testing.

	Relevant Findings
Bone marrow morphological evaluation	Infiltration by monoclonal lymphoplasmacytic cells (>75%) and small plasma cells with lambda restriction.
Bone marrow flow cytometry	(1) B-cell population: CD45++, CD19+, CD20−, CD22++, CD25+, CD10−, CD5−, CD38−, BCL2+ (2) Plasma cell population: CD19+, CD38++, CD20−, CD25−, CD45+
Histology	Lymphoid neoplastic population with a diffuse pattern with more than 75% of tumor cells with scattered plasma cells and increased reticulin deposition.
Molecular testing	MYD88^L265P^ mutation CXCR4^S342^mutation

## Data Availability

Data sharing is not applicable to this article.
